# Orthodontic treatment of unilateral cleft lip and palate associated with maxillary canine/premolar transposition: case report

**DOI:** 10.1590/2177-6709.25.3.054-064.oar

**Published:** 2020

**Authors:** Rodrigo Matos de Souza, Henrique Telles de Oliveira, Marcel Marchiori Farret

**Affiliations:** 1 Fundação para Reabilitação das Deformidades Crânio-Faciais, Departamento de Ortodontia (Lajeado/RS, Brazil).

**Keywords:** Dental occlusion, Cleft lip, Orthodontic space closure

## Abstract

**Introduction:**

The cleft lip and palate is the most frequent craniofacial anomaly and as a consequence of this malformation some inadequate occlusal relationship between the arches are observed. Furthermore, dental absences, individual positioning changes of teeth as rotations, and in more rare situations the transpositions may be found as well.

**Description:**

In this context, in this article is reported a case of a 9-year-old patient with unilateral cleft lip and palate, with anterior and posterior crossbite on the left side, absence of the maxillary left lateral incisor, and transposition of the maxillary left canine and first premolar. The patient was treated with slow maxillary expansion, secondary graft and fixed orthodontic appliance, transposition maintenance and closing of the lateral incisor space with the first premolar, by means of mesialization of the posterior teeth.

**Results:**

At the end of the treatment, good intercuspation and an important aesthetic gain for the patient were achieved. The analysis three years after treatment revealed a good stability of the results obtained.

## RESUMO

**Introdução:**

A fissura labiopalatina é a anomalia craniofacial mais frequente e, como consequência dessa malformação, observa-se normalmente uma relação oclusal inadequada entre as arcadas. Além disso, nesse pacientes também podem ser observadas ausências dentárias, alterações de posicionamento individual dos dentes, como rotações e, em situações mais raras, as transposições também podem ser encontradas.

**Descrição:**

Neste contexto, é relatado neste artigo o caso clínico de um paciente de 9 anos de idade com fissura labial e palatina unilateral, com mordida cruzada anterior e posterior no lado esquerdo, agenesia do incisivo lateral superior esquerdo e transposição do canino com o primeiro pré-molar superior esquerdos. O paciente foi tratado por meio de expansão maxilar lenta, enxerto ósseo secundário e aparelho ortodôntico fixo, aceitando-se a transposição e fechando o espaço da agenesia do incisivo lateral com o primeiro pré-molar posicionado nesse espaço, seguido de mesialização dos dentes posteriores.

**Resultados:**

Ao fim do tratamento, obteve-se boa intercuspidação e importante ganho estético para o paciente. A análise três anos após o tratamento revelou uma boa estabilidade dos resultados obtidos.

## INTRODUCTION

The cleft lip and palate is a congenital malformation characterized by the absence of fusion of the palatine processes during the embryonic phase, with high prevalence, present in 1 in 1,100 births in the world, being the more frequent craniofacial anomaly.^1^ ^-^ ^6^ Clinically the clefts are classified according to the incisive foramen, and divided into four types: pre-foramen clefts or lip cleft, post-foramen clefts or palate cleft, transforaminal cleft or cleft lip and palate, and the rare fissures of the face.^6^ In addition, fissures can be found unilaterally, bilaterally or medial, with unilateral clefts being the most frequent.^2^ ^,^ ^7^

In the region of the cleft, it is common to observe problems of occlusal relationship such as posterior or anterior crossbite due to contraction of the upper arch, absence of permanent lateral incisor, rotations, changes of crown shape and, in some situations, the dental transpositions.^1^ ^,^ ^3^ ^,^ ^8^ ^-^ ^12^

The dental transposition is considered a subdivision of the ectopic eruption, and is an order or position disturb with prevalence of 0.4% in the population. However, its prevalence in patients with cleft lip and palate is considerably higher, around 14%.^11,13-15^ It is characterized by the change of position of two adjacent teeth in the dental arch, in the same quadrant, and its etiology is still not fully understood: recent evidence points to multifactorial hereditary genetic influence due to the bilateral occurrence of the problem.^7^ ^,^ ^8^ ^,^ ^11^ ^,^ ^14^ ^,^ ^16^ The transposition treatment is based primarily on the decision to accept or correct the transposition and based on that, depends on several factors such as occlusal relationship in the maxillary and mandibular arches, alveolar bone thickness, individual tooth positioning, age of the patient, inherent risks such as resorptions, gingival recession and fenestration, aesthetic characteristics of the smile, among others.^7,8,16,17^ Furthermore, another important decision in cases of cleft lip and palate associated to lateral incisor absence is to keep the space for rehabilitation or close the space through the mesial movement of posterior teeth along the bone graft. This decision must be based on many factors as the age of the patient, occlusal relationship, bone condition, smile esthetics and mainly the long-term functional and esthetic result.^1^ ^,^ ^2^ ^,^ ^4^ ^-^ ^6^

Based on that, this manuscript presents a case of a patient with left unilateral transforaminal cleft, with anterior and posterior crossbite, lateral incisor absence and transposition between canine and first premolar. The patient was treated with slow maxillary expansion and orthodontic mechanics to close the spaces through the mesialization of the posterior teeth, accepting the transposition and positioning the upper left first premolar at the place of the absent left lateral incisor.

## CASE REPORT

The patient, a 9-year-old boy, sought for treatment at the Foundation for Rehabilitation of Craniofacial Deformities (FUNDEF) in Lajeado (RS, Brazil), due to the presence of cleft lip and palate.

### Diagnosis

**Figure 1:**
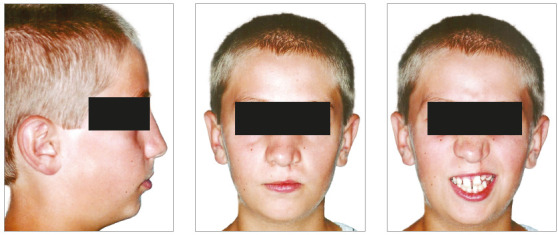
Initial facial photographs.

**Figure 2:**
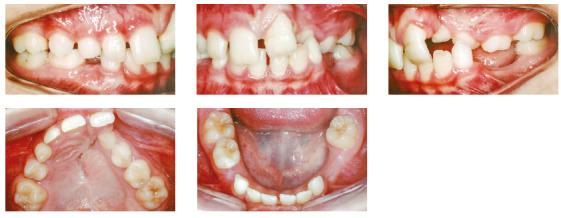
Initial intraoral photographs.

**Figure 3:**
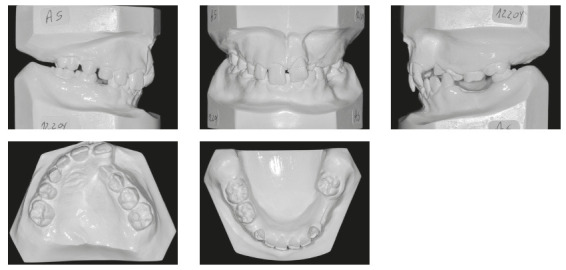
Initial dental casts.

**Figure 4:**
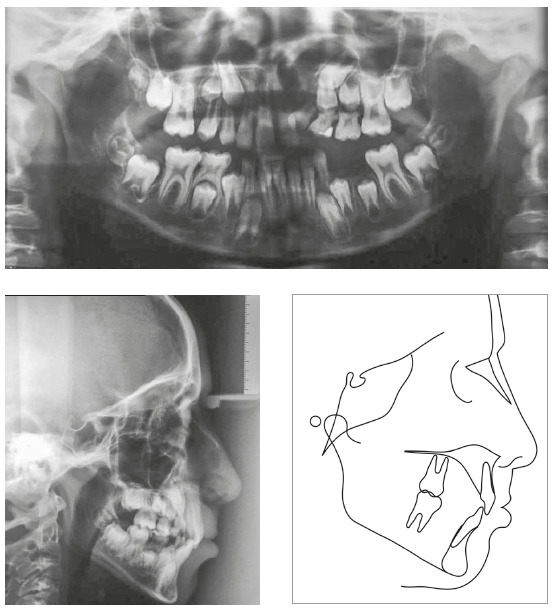
Initial radiographies and cephalometric tracing.

In the analysis of the initial facial photographs, it was possible to verify that the patient had proportional facial thirds, passive lip seal, convex profile and discreet flattening of the left nostril and lip asymmetry on the left, as a result of the unilateral cleft and consequently of the primary surgeries of cheiloplasty and palatoplasty, which had been performed in the months following his birth (Fig 1). In the intraoral and dental casts analysis, it was verified a Class II molar relationship on both sides, absence of the left maxillary left lateral incisor, deviation of 2mm between the maxillary and mandibular midlines, with the maxillary midline matching the facial midline (Figs 2 and 3). In addition, clinically there was also anterior and posterior crossbite on the left side as a result of lack of transverse development of the maxillary arch in that region. Through the panoramic radiograph, the absence of the maxillary left lateral incisor was confirmed, all other permanent teeth were present, except for the germ of the tooth #28 and the complete transposition of the maxillary left canine with the first premolar was also identified in the same quadrant. Lateral cephalogram along with the cephalometric tracing showed a facial pattern with increased vertical growth, skeletal Class II, maxillary incisors uprighted and mandibular incisors proclined (Fig 4). 

### Treatment objectives

The objectives of the treatment were to:

1. Correct the anterior and posterior crossbite.
2. Perform secondary alveolar bone grafting after slow maxillary expansion.
3. Accept transposition due to the risks of attempted correction
4. Close the space of the maxillary lateral incisor absence with the first premolar, through the mesialization of the posterior teeth.
5. Perform rehabilitation procedures on the anterior teeth after orthodontic treatment

### Treatment plan

Initially, a slow maxillary expansion was planned to be performed with a fixed quadrihelix. After the end of the expansion, the autogenous secondary graft removed from the iliac crest would be performed and the upper and lower fixed orthodontic appliances would be bonded. After alignment and leveling, the right maxillary second premolar extraction would be done and a mini-implant would be installed in the maxillary left quadrant, to assist the anchorage in the mesialization of the posterior teeth of this side, to close the space of the absence of the maxillary left lateral incisor. In the finishing procedures, bending and rebondings would be planned to optimize the positioning of the maxillary anterior teeth and later the patient would be referred for aesthetic restorations.

### Treatment alternative

The alternative considered for this case was the distalization of all teeth on the left side of the maxillary arch, thus opening space for rehabilitation. The main advantage of this option would be the reduction of the treatment time, because probably the distalization would be faster than the mesialization over the bone graft region. However, one important disadvantage would be bone graft condition and the aesthetic and functional result of the rehabilitation, because an implant would have doubtful success rate and longevity, and a conventional prosthesis would not maintain the amount and quality of the bone in long-term. Furthermore, the slow mesial movement of the posterior teeth would have a beneficial effect over the bone graft on that region. 

### Treatment progress

**Figure 5:**
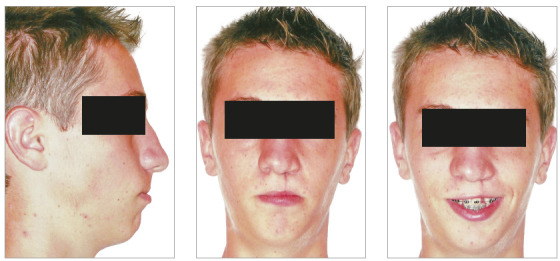
Facial photographs during the progress of treatment.

**Figure 6:**
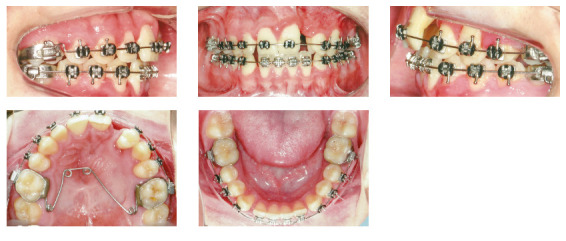
Intraoral photographs during the progress of treatment, after the expansion with quadrihelix and in the beginning of the alignment and leveling.

**Figure 7:**
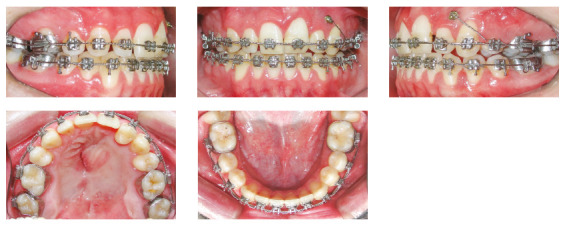
Intraoral photographs during the progress of treatment, after closing spaces on the left side of the maxillary arch. Mini-implant remained connected to the teeth, to keep the space closed.

**Figure 8:**
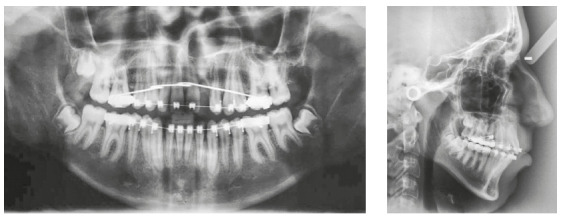
Radiographies during the progress of treatment.

**Figure 9:**
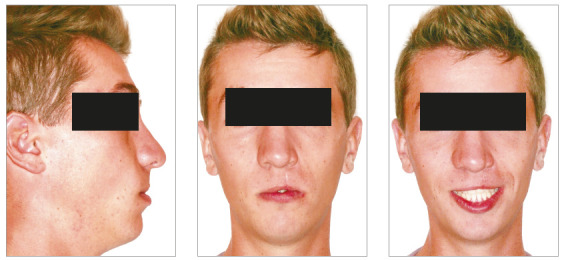
Posttreatment facial photographs.

The treatment began at 10-years old with the installation of a quadrihelix appliance in the maxillary arch for slow expansion of the maxilla, with activation once a month, for 8 months. After expansion, the device was kept in position for another 12 months and during this period of retention, the patient was referred to perform a secondary autogenous graft from the iliac crest, performed by the FUNDEF team of surgeons, at the Bruno Born Hospital. At 12-years-old, 0.022 x 0.028-in Roth brackets were bonded, and the alignment and leveling was performed initially with round 0.012-in and 0.014-in NiTi wires, followed by 0.016-in to 0.020-in stainless steel archwires, and 0.018 x 0.025-in rectangular stainless steel archwires. The quadrihelix was kept in position during the first stage of alignment and leveling, and was replaced soon after by a transpalatal bar After alignment and leveling, the maxillary right second premolar was extracted and the space was almost totally occupied by the eruption of the first premolar on that region, leaving a small space to be closed by mesialization of the posterior teeth. To assist in the mesialization of the upper left posterior teeth, a mini-implant was installed between the roots of teeth #23 and #24. The mini-implant was connected to the second molar by 0.012-in braided metallic ligation and the teeth were mesialized individually, with open coils with light forces (around 60-80 g/f), initially positioned between the teeth #23 and #24, being transferred to distal after the closing of the anterior spaces. In the finishing phase, palatal torque and extrusion bend were performed on tooth #24, with the aim of hiding the root volume and moving the gingival contour in an incisal direction in this tooth, which was positioned in place of the lateral incisor. Furthermore, the buccal and lingual cusps were worn to allow the extrusion and to avoid interferences. After seven years of treatment (two years of expansion and retention + five years of corrective orthodontics) the appliance was debonded, and a 3 x 3 mandibular fixed retainer and maxillary removable wraparound were installed (Figs 5 to 9). 

### Treatment results

**Figure 10:**
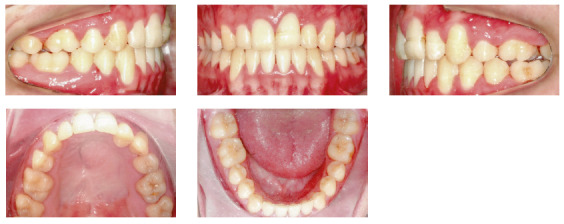
Posttreatment intraoral photographs.

**Figure 11:**
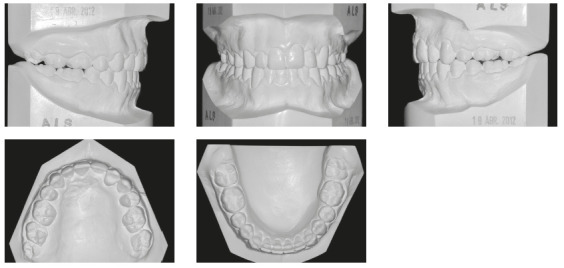
Posttreatment dental casts.

**Figure 12:**
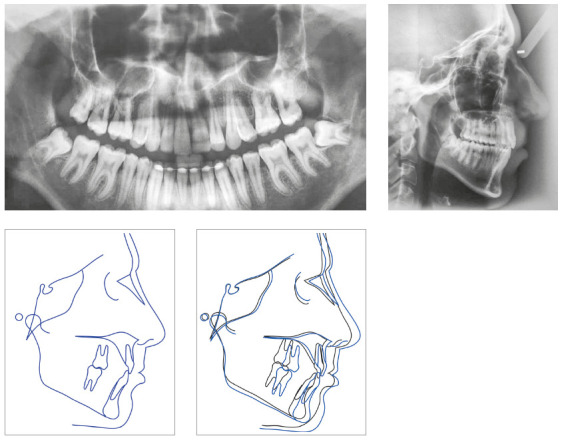
Posttreatment radiographies, Posttreatment cephalometric tracing and total superimposition.

**Figure 13:**
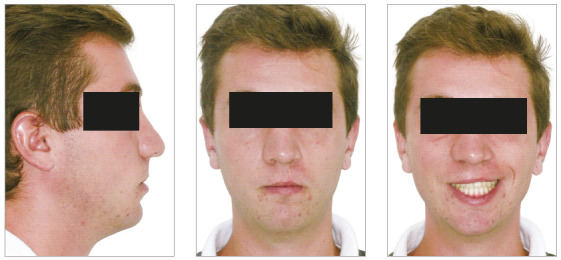
3-year follow-up facial photographs.

**Figure 14:**
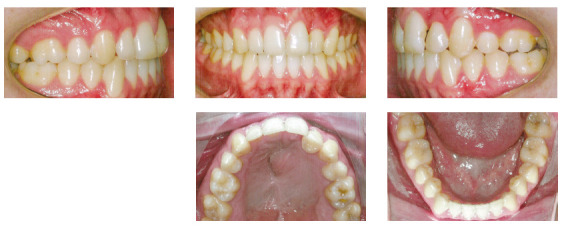
3-year follow-up intraoral photographs.

At the end of the treatment, it was possible to observe the preservation of the facial aspects, with a significant improvement in the aesthetics of the smile (Fig 10). In the intraoral analysis, it was verified that a coincidence was established between the midlines, molar Class II and canine Class I relationship were obtained, with good intercuspation, adequate overjet and overbite, and the space of the upper left lateral incisor was successfully closed by the premolar replacement, obtaining good aesthetics and adequate function by the anterior and lateral guidances (Figs 11 and 12). In the panoramic radiograph, a good root parallelism was evident, except for the tooth #14; moreover, a slightly root apical resorption in the maxillary and mandibular anterior teeth was observed. In the lateral cephalogram, cephalometric analysis and superimpositions, a small improvement in the anteroposterior skeletal pattern was observed, an small change in the maxillary and mandibular incisors position and accentuated molar mesialization on the maxillary arch (Fig 13 and Table 1). In the 3-year follow-up analysis, it was possible to verify that the results obtained were stable, with a good occlusal relationship and preservation of the health of the tissues (Fig 14).

**Table 1:** Cephalometric measurements.

Measurements	Normal	Initial	Posttreatment
SNA	82^o^	82^o^	80^o^
SNB	80^o^	72^o^	74^o^
ANB	2^o^	9^o^	6^o^
Facial convexity (NA.APog)	0^o^	17^o^	12^o^
Facial angle (PoOr.NPog)	87^o^	82^o^	84^o^
Y-axis	59^o^	64^o^	62^o^
SN.GoGn	32^o^	42^o^	40^o^
1.NA (degrees)	22^o^	1,5^o^	2^o^
1-NA (mm)	5mm	-4mm	-2mm
1.NB (degrees)	25^o^	30^o^	17^o^
1-NB (mm)	5mm	7mm	6mm
Interincisal angle	131^o^	141^o^	153^o^
Ul-S line	0mm	0mm	-2mm
Ll-S line	0mm	5mm	2mm
IMPA	90^o^	97^o^	85^o^
FMA	25^o^	32^o^	31^o^
FMIA	65^o^	51^o^	64^o^

## DISCUSSION

Patients with cleft lip and palate always represent a challenge for the orthodontist, due to the complexity of the mechanics involved in the correction of asymmetries, elimination of crossbite, correction of individual dental positions and closure of spaces of missing teeth. Absence of the lateral incisor is often associated with cleft lip and palate, and the orthodontist along with the rehabilitation team should define the best treatment option in this region.^1^ ^,^ ^2^ ^,^ ^4^ ^-^ ^6^ ^,^ ^18^ Even after a successful secondary graft in the cleft region, there is a tendency to remain a vertical defect in this region, which in most situations contraindicates rehabilitation through implant and prosthesis.^2,5,19^ In addition, implant and prosthesis rehabilitation in the long term tends to have a greater aesthetic compromise due to non-vertical physiological migration of the implant, which would imply the need for a new rehabilitation to restore vertical symmetry in this region.^5^ ^,^ ^19^ Thus, two options are usually considered: space closure with orthodontic movement, or rehabilitation with conventional prostheses. When the secondary graft performed after the expansion provides a good amount of alveolar bone in the bucco-lingual and vertical direction, and the posterior teeth show adequate root condition, it is recommended to close the space, avoiding the necessity for rehabilitation at the end of the treatment, being necessary only an aesthetic adequacy of the anterior teeth that will occupy the space of adjacent absent teeth.^19^ In the case reported, despite the presence of transposition, the posterior teeth showed adequate condition for the mesial movement and, in addition, excellent bone quantity and quality were obtained after the secondary graft, also favoring the closure of the space by orthodontic movement.

The decision to accept or correct a transposition is based on several factors, and must be made so that the benefits to the patient outweigh the harm.^7^ ^,^ ^8^ ^,^ ^11^ ^,^ ^17^ The correction attempt should be made in adequate bucco-lingual thickness in the region, the integrity of periodontal tissue in the teeth involved, the presence of all teeth in the quadrant and also in situations of great aesthetic damage by transposition.^11^ ^,^ ^13^ ^-^ ^16^ ^,^ ^20^ In the case presented, the patient already had lateral incisor absence in the maxillary left quadrant, associated with loss of bone tissue inherent to cleft lip and palate situations, so the attempt to correct the transposition could represent a risk of loss or impairment of one or more teeth during mechanics, increasing the aesthetic and functional impairment for the patient. In addition, from a functional point of view, with the acceptance of the transposition, a Class I canine relationship would be established. With the correction of the transposition, the premolar should perform the canine function, compromising the occlusal function. From an aesthetic standpoint, the transposition correction would take the canine to the lateral incisor site and the first premolar to the canine site, making it even more difficult to obtain adequate aesthetics in the anterior region.

Positioning of a premolar in the place of a upper lateral incisor may represent an aesthetic problem due to the difference in the shape of the crown between these two teeth, being the premolar more convex in the buccal surface and having more parallel mesial and distal surfaces between them.^1^ This problem can be overcome with wear and/or restorations. In addition, the presence of the palatal cusp of the premolar may cause interference during the anterior and lateral guidance movements, and should be worn to avoid this contact.

Some limitations imposed by the occlusion and periodontal tissues of the cleft patients prevent better aesthetic and functional results and in shorter periods of time.^1^ Usually these patients have little motivation and little collaboration with the treatment, with the use of elastics, hygiene and other necessary care with the appliance during the treatment.^5^ However, even with these limitations, at the end of the treatment, adequate aesthetics and function were obtained and in the analysis three years after treatment it was possible to verify the stability of the obtained results.

## CONCLUSION

Based on the literature review and on the results observed in the reported case, it is possible to affirm that the acceptance of the transposition of teeth in a region of cleft lip and palate is an adequate option from the standpoint of preservation of the teeth and periodontal tissues of the region, and allows to obtain good esthetic and functional results, even in long term.
